# Effects of the COVID-19 Pandemic on the Perception of Inclusion in School Education and Physical Activity Among Polish Students

**DOI:** 10.3389/fpsyg.2022.880791

**Published:** 2022-07-26

**Authors:** Karolina Kostorz, Anna Zwierzchowska, Mateusz Ziemba

**Affiliations:** ^1^Jerzy Kukuczka Academy of Physical Education in Katowice, Katowice, Poland; ^2^WSB University in Poznan, Faculty in Chorzow, Chorzow, Poland

**Keywords:** school, students, adolescents, COVID-19, physical activity, perception of inclusion, pupils

## Abstract

The coronavirus disease-2019 (COVID-19) pandemic impacted the lives of children and adolescents, leading to many changes in their routines, especially in education. Face-to-face physical education (PE) classes during COVID-19 were affected in organization, possibly conditioning students' participation, motivation, and learning. In the extreme conditions of the coronavirus, it may be assumed that daily physical activity became much less than before, partly because students are learning outside the school environment and PE lessons taught using remote forms do not fulfill their purpose. The aim of the study was to assess the pupil's perception of inclusion in school education during the remote learning caused by the COVID-19 pandemic and to compare the results with the control group. Moreover, the physical activity of respondents during social isolation due to the coronavirus was examined. The sample consisted of 111 pupils of both genders, aged 14–21 years. The Perceptions of Inclusion Questionnaire (PIQ) was used to measure the perception of inclusion in school education. The structure of the participants' physical activity was examined using the International Physical Activity Questionnaire-Long Form (IPAQ-LF) for adolescents. It was observed that the pupils' gender did not differentiate their perception of inclusion in school education. It was proved that respondents participating in research during the COVID-19 pandemic obtained statistically significantly lower results in the case of “emotional wellbeing in school” (*p* < 0.001; η^2^ = 0.07) but a higher mean was observed in relation to “social relationships with other pupils” (*p* = 0.04; η^2^ = 0.02) than the control group. Girls achieved a higher mean in the case of walk Metabolic Equivalent of Task (MET) (*p* = 0.02; η^2^ = 0.06) than boys. In addition, it was observed that the recommendation of vigorous physical activities was achieved by 37.78% of boys and 34.85% of girls. In turn, 69.70% of female pupils and 77.78% of male respondents met the recommendations for medium physical activities. It was also noted that 87.88% of girls and 86.67% of boys participating in the research achieved the recommendation for total physical activities. The analysis showed negligible and low positive correlations between examined variables.

## Introduction

### Inclusion in Education

The main task of school should be the inclusion of each student, regardless of gender, age, place of residence, race, poverty, degree of disability, ethnicity, indigeneity, wealth, language, religion, migration or displacement status, sexual orientation or gender identity and expression, incarceration, beliefs, and attitudes in the education process (Qvortrup and Qvortrup, 2018; Messiou, 2019; Messiou and Ainscow, 2020; Schwab et al., 2020; UNESCO, 2020). Successful inclusive education is a process of strengthening the capacity of the education system to create a learning environment for the achievement of high-quality education that supports the cognitive abilities of all learners and their social and emotional development (UNESCO, 2009; Qvortrup and Qvortrup, 2018; Antoninis et al., 2020; Fernández-Archilla et al., 2020; Głodkowska, 2020).

### Physical Activity and E-Inclusion During the COVID-19 Pandemic

It is pointed out that the situation caused by the coronavirus disease-2019 (COVID-19) pandemic has become a public health threat to people around the world, affecting their lifestyle and mental health (Clemente-Suárez et al., 2020; Mavilidi et al., 2020; Di Stefano et al., 2021). Wang et al. (2020) indicated that when children are locked in their homes without outdoor activities and interaction with their friends, it may have negative effects on their wellbeing, health, and perception of inclusion in the school education process.

It is, therefore, worth considering how online learning affects interpersonal relationships and interactions and the social field, i.e., students' perception of inclusion in school education. It seems that, on the one hand, the Internet can support the online learning process, but, on the other hand, it can be a factor that negatively affects students' perception of inclusion in school education. Twenge et al. (2019) noticed that many mental illnesses, such as depression and addiction, have escalated with the increasing smartphone and social network use, and this is undoubtedly observed during the coronavirus pandemic. Moreover, Biddle and Asare (2011), on the basis of a literature review, indicated that screen time is negatively associated with young people's mental health. Hoare et al. (2016) emphasized that leisure screen-based sedentary behaviors are associated with higher psychological distress and lower self-esteem. The social isolation caused by the COVID-19 pandemic is an anomalous situation that obliges people, including students, to modify their normal life habits and daily routines (Clemente-Suárez et al., 2020; Girdhar et al., 2020; Venkatesh and Edirappuli, 2020; Di Stefano et al., 2021; Mata et al., 2021). These extreme conditions could negatively affect adolescents' relationships, perception of inclusion in the school education, and social connections (Girdhar et al., 2020; Lebel et al., 2020), especially when they did not previously have good relationships with their relatives (Rosen et al., 2020). For several years, authors have argued that striving to strengthen the students' perception of inclusion in school education is one of the fundamental missions and that it is very important to conduct research exploring the predictors of this construct among students around the world (Ahmadi and Ahmadi, 2020).

In view of the above, it seems that physical education (PE) at school is one of the elements that affect students' perception of inclusion in the school environment, their self-esteem, and wellbeing (Hasanpour et al., 2014; Goldfield et al., 2015; Fernández-Bustos et al., 2019; Mata et al., 2021). Polak and Tarkowski (2020) emphasized that participation in PE classes can shape one of the most important social values, which is solidarity, can be a source of lasting friendships, can teach loyalty and brotherhood, and can develop the ability to cooperate in a group. Moreover, Mavilidi et al. (2020) reported that physical activity could be beneficial for students' cognition, meta-cognition, engagement, and academic performance. In addition, Lubans et al. (2016) and Rodriguez-Ayllon et al. (2019) noticed that owing to higher levels of physical activity and sedentary behavior reduction, basic psychological needs (e.g., social relationships, self-acceptance, and perception of purpose in life) can become satisfied and mental health can be enhanced in children and adolescents. Therefore, on the basis of the reports mentioned earlier, it can be assumed that the issues of perception of inclusion and physical activity are separated but essentially interrelated the aspects that condition students' lives with reference to more positive psychological functioning.

Certainly, during the COVID-19 pandemic, a sedentary lifestyle predominates among adolescents and students (Di Stefano et al., 2021; Mata et al., 2021). It may be assumed that in these extreme conditions, daily physical activity is much lower than before, partly because students are learning outside the school environment and PE lessons taught by using remote forms do not fulfill their activating purpose (Di Stefano et al., 2021). In addition, it seems that organizing PE lessons outside the gym and without direct contact between the teacher and the students, as well as among peers, can exert an extremely negative impact on the perception of inclusion in the school education process (Mata et al., 2021).

However, Tang and Ying (2020) reported that although 87% of students in the world have been physically, socially, and psychologically affected by school breaks caused by the pandemic, there are still no thorough studies on their mental health in these settings. According to the available research results, during out-of-school periods (e.g., weekends or holidays), students become less physically active, engage in much longer screen time, develop irregular sleep patterns, and indulge in less healthy diets, which leads to overweight and decreased cardiorespiratory fitness (Brazendale et al., 2017; Wang et al., 2019). Although students' socio-emotional development (e.g., social inclusion, school wellbeing, and academic self-concept) has gained more and more attention over the past decades (Schwab et al., 2013, 2018, 2020), there are no publications available concerning the effects of social isolation and learning online caused by the COVID-19 pandemic on the perception of being included in school education. This was an inspiration to conduct the present research. Being aware of that the lack of school inclusion and physical activity, not only during a pandemic, are very important issues, we decided to conduct our investigation in this area.

### Objectives

The aim of the study was to assess students' perception of inclusion in school education during the COVID-19 pandemic distance learning period in comparison with the control group. Moreover, the physical activity of the respondents during social isolation was examined. With the consideration of other authors' publications, the following research questions were formulated:

- Do the respondents meet the World Health Organization's (WHO) global recommendations for children's and adolescents' PA?
- Is remote learning as a consequence of the COVID-19 pandemic a differentiating factor when it comes to the students' perception of inclusion in the education process?
- What are the relationships between the students' perception of inclusion in the education process and other studied variables, i.e., PA, gender, and body mass index (BMI) index?
- Is gender a differentiating factor in relation to the students' PA level and perception of inclusion in the education process?

## Materials and Methods

### Participants

The research was conducted in the years 2020–2021 in the Silesian Voivodeship and the Warmian-Masurian Voivodeship. Non-probability consecutive sampling was applied in the study, and participation was voluntary. All individuals gave written consent to voluntarily participate in the research. In the case of minors, consent was obtained from the parents or legal guardians. The sample consisted of 111 students of both genders, aged 14–21 years (*M* = 16.48; *SD* = 1.48; *Mo* = 17; *Me* = 16.4; V = 9%) and attending mass schools. The obtained results were compared with the data collected in the years 2018–2019 among 112 students aged 10–16 years attending mass, integrated, and segregated schools.

### Measurements and Procedures

The diagnostic poll method with the questionnaire technique was used to fulfill the assumed aims. Standardized research tools were applied.

The Perceptions of Inclusion Questionnaire (PIQ) (Zurbriggen et al., 2017) was used to measure the perception of inclusion in school education in Polish students. The tool was downloaded from its dedicated website.^1^ PIQ contains 12 statements. It is worth adding that the items are short and use accessible wording. The task of the students was to rank the particular statements using a 4-point Likert scale, where 1 meant “not at all true,” 2 meant “somewhat not true,” 3 meant “somewhat true,” and 4 meant “certainly true.” Reverse coding was applied for the negatively formulated items, i.e., items 4, 8, and 12. Subscale scores were obtained by summing the ratings of the four items on each scale. The tool addresses three relevant issues, namely, the student's perception of (1) emotional wellbeing in school, (2) social relationships with other students, and (3) academic competencies.

In turn, the structure of the participants' physical activity was examined with the International Physical Activity Questionnaire-Long Form (IPAQ-LF) (Craig et al., 2003) for adolescents. IPAQ was developed for surveillance activities and to guide policy development related to health-enhancing physical activity across various life domains, in response to the global demand for comparable and valid measures of physical activity within and between countries. The research tool that was mentioned earlier has been developed to estimate the levels of habitual physical activity across different countries and sociocultural environments. The questionnaire consists of five independent parts including 27 questions. The questions refer to all possible physical activities undertaken within the previous 7 days. The research tool can be downloaded from the website.^2^ IPAQ-LF measures physical activity (in MET-minutes/week units) in various domains of everyday life, including at work (or at school), while traveling, doing housework or leisure activities, and sports (Craig et al., 2003). The obtained data are used to estimate total weekly physical activity by weighting the reported minutes per week within each activity category by aMET energy expenditure estimate assigned to each category of activity. MET levels were obtained from the 2,000 compendium of physical activities to include moderate-intensity activities between 3 and 6 METs and vigorous-intensity activities as >6 METs. The weighted MET-minutes per week (MET·min·wk^−1^) were calculated as duration *x* frequency per week *x* MET intensity, which was summed across activity domains to produce a weighted estimate of total physical activity from all reported activities per week (MET·min·wk^−1^) (Craig et al., 2003). The exact figures for MET values for each domain and intensity can be found on the website.^3^ In this paper, it was explored whether the respondents met the global recommendations on physical activity for young people as presented in the guidelines of the WHO (2018).

Members of the school administration, parents, and students were informed about the aim of the study. The diagnostic questionnaires and a list of demographic questions, which allowed us to obtain data on the respondents' age, gender, and place of residence, were made available remotely by using the European Indares^4^ (International Database for Research and Educational Support) platform. The researchers provided instructions on how to correctly complete the questionnaire. The results presented in the manuscript are a part of the project entitled “Physical condition and motor skills of children and adolescents in the face of the perception of inclusion in school education.” The research was conducted in the years 2018–2020 and funded by the Ministry of Science and Higher Education as part of the statutory research of the Jerzy Kukuczka Academy of Physical Education in Katowice. The study was conducted in accordance with the principles of the Declaration of Helsinki and received ethical approval from the Bioethics Committee of the Academy of Physical Education in Katowice (Resolution No. 2/2018 as of 21 June 2018).

### Data Analyses

Microsoft Office Excel 2010, StatSoft Statistica version 13, and JASP version 0.14.1 software were used for the purposes of statistical analyses.

According to the data available on the website of the Republic of Poland, the authorities of the Silesian Voivodeship report that about 160,961 children and adolescents attended secondary schools in the 2019/2020 school year. Therefore, it was calculated that 384 was the minimum recommended sample size for the study. At the same time, it turned out that for the sample of 111 respondents, the margin of error was 9.30%. It should be added that in the case of 111 students participating in the research, a confidence level above 71% was obtained.

The basic analyses of the data employed descriptive statistics for the whole sample and for the population stratified by gender. The distribution was tested for normality with the Shapiro–Wilk *W* test. Levene's test was used to assess the equality of variances. One-way ANOVA was used to verify the significance of differences between the tested variables. The significance level was assumed at *p* < 0.05. Practical significance was evaluated with the eta squared (η^2^). The small effect size was assumed for values ranging from 0.01 to 0.05, moderate for values ranging from 0.06 to 0.13, and large for η^2^ ≥ 0.14 (Richardson, 2011). Frequency tables allowed for assessing the meeting of the WHO (2018) global recommendations on physical activity. Pearson's linear correlation coefficient was used to calculate the strength of the associations between the investigated variables. A negligible correlation was assumed for values ranging from 0.0 to 0.3, low for values ranging from 0.3 to 0.5, moderate for values ranging from 0.5 to 0.7, high for values ranging from 0.7 to 0.9, and very high for values ranging from 0.9 to 1.0 (Mukaka, 2012).

## Results

In the first step of the analysis, students' perception of inclusion in school education during the COVID-19 pandemic was assessed with the consideration of the respondents' gender (Table 1).

**Table 1:** Analyses of differences in the PIQ's results between girls and boys.

**Studied variable**	**Girls**		**Boys**		**Error: between MS**	**df**	***p*-value**	***η^2^***
	***M***	***SD***	***M***	***SD***				
Emotional wellbeing in school	10.15	2.78	9.60	3.12	8,54	109	0.37	0.01
Social relationships with other pupils	11.74	2.38	12.40	2.86	6,67	109	0.23	0.02
Academic competencies	10.33	2.25	11.09	2.64	5,82	109	0.14	0.03
Sum PIQ score	32.23	5.92	33.09	6.16	36,25	109	0.50	0.01

The presented data imply that the students' gender did not differentiate their perception of inclusion in school education.

In the second step of the analysis, the results of the respondents were compared with the control group (Table 2).

**Table 2:** Comparison of the PIQs' results collected during the COVID-19 pandemic in 2021 with the control group.

**Studied variable**	**Results obtained during the pandemic in 2021**		**Control group**		**Error: between MS**	**df**	***p*-value**	***η* ^2^**
	***M***	***SD***	***M***	***SD***				
Emotional wellbeing in school	9.93	2.92	11.30	1.85	5.97	221	<0.001	0.07
Social relationships with other pupils	12.01	2.59	11.43	1.82	5.02	221	0.04	0.02
Academic competencies	10.64	2.43	10.58	1.68	4.36	221	0.83	<0.01
Sum PIQ score	32.58	6.01	33.31	3.68	24.77	221	0.27	<0.01

It turned out that during the COVID-19 pandemic, the research participants obtained statistically significantly lower results in the case of “emotional wellbeing in school” (*p* < 0.001), but a higher mean was observed for “social relationships with other students” (*p* = 0.04) as compared with the adolescents tested in 2018–2019. What draws attention is the effect size, which was moderate with regard to “emotional wellbeing in school” (η^2^ = 0.07) and small for “social relationships with other students” (η^2^ = 0.02).

The following analyses considered the level of students' physical activity during the COVID-19 pandemic (Table 3).

**Table 3:** Analyses of differences in the IPAQ-LF's results between girls and boys.

**Studied variable**	**Girls**		**Boys**		**Error: between MS**	**df**	***p*-value**	***η* ^2^**
	***M***	***SD***	***M***	***SD***				
Job-related activities MET	1,209.10	2,160.88	1,280.86	2,115.91	4592E3	109	0.87	<0.01
Transportation MET	1,098.91	1,181.10	1,470.70	2,100.96	2614E3	109	0.28	0.01
Housework MET	1,021.89	1,823.29	1,106.50	2,010.26	3614E3	109	0.83	<0.01
Sports and recreational activities MET	1,046.58	1,319.15	1,331.47	1,747.40	2270E3	109	0.37	<0.01
Vigorous MET	817.73	1,525.73	1,477.33	2,343.48	3605E3	109	0.10	0.03
Medium MET	1,708.26	2,028.61	2,720.72	3,318.21	6899E3	109	0.07	0.03
Walk MET	1,850.50	1,959.04	991.47	1,184.94	2855E3	109	0.02	0.06
SUM MET	4,376.48	3,669.55	5,189.52	5,348.28	1958E4	109	0.39	0.01

It was revealed that only the level of walk MET (*p* = 0.02) differentiated girls and boys. Higher results were reported among female students. It is worth noting that the effect size was moderate (η^2^ = 0.06).

In addition, it was observed that the recommendations for vigorous physical activity were met by 37.78% of boys and 34.85% of girls. In turn, 69.70% of female students and 77.78% of male respondents met the recommendations for medium physical activity. It was also noted that 87.88% of girls and 86.67% of boys participating in the research fulfilled the recommendations for total physical activity.

An important aspect of the research was also to answer the following question: Are there any correlations between the students' perception of inclusion in school education and their physical activity or BMI, and are there any differences related to the respondents' gender in this respect? To find the relevant results, Pearson's linear correlation coefficient was applied. The obtained statistically significant values (*p* < 0.05) are presented in Table 4.

**Table 4:** Correlation coefficients.

	**Studied variable**	**Emotional wellbeing in school**	**Social relationships with other**	**Academic competencies**	**Sum PIQ score**
Whole sample	BMI index			0.3	
	Vigorous MET			0.2	0.2
Girls	BMI index			0.3	
Boys	Housework MET		0.3		0.4
	Medium MET			0.3	0.3

It is surprising that in the case of the whole sample, the analyses revealed a low positive correlation between BMI and “academic competencies” (*r* = 0.3) and a negligible relationship between “vigorous MET” and “academic competencies” (*r* = 0.2), as well as between “vigorous MET” and “sum PIQ score” (*r* = 0.2). Among girls, only one low positive correlation was observed between BMI and “academic competencies” (*r* = 0.2). Besides, in the male group, “housework MET” presented a low positive relationship with “social relationships with other students” (*r* = 0.3) and “sum PIQ score” (*r* = 0.4). In addition, “medium MET” exhibited a low positive correlation not only with “academic competencies” (*r* = 0.3) but also with “sum PIQ score” (*r* = 0.3).

## Discussion

The WHO (2020) announced the novel COVID-19 as a public health emergency. Poland and other countries had to implement unprecedented means to prevent the spread of the disease. Governments around the world decided that both indoor and outdoor sports and recreational facilities were closed (Giustino et al., 2020; Shahidi et al., 2020; Wang et al., 2020; Di Stefano et al., 2021; Gjaka et al., 2021). Di Stefano et al. (2021) indicated that the restrictions taken to control the rapid spread of COVID-19 resulted in a sudden, unprecedented change in people's lifestyles and habits, leading to several negative consequences on general health. It was found that during social distancing, 28% of the respondents reported a headache worsening, 33% reported an improvement, and 39% reported a stable headache frequency (Di Stefano et al., 2021). A significant decrease in the PA levels during COVID-19 quarantine and the sleep quality in migraine was observed (Di Stefano et al., 2021). Of course, the closure of indoor and outdoor sports and recreational facilities was a very important issue for children, who should participate in and enjoy physical activity during their leisure time as part of a broader set of life skills. The closure of school may cause disruptions not only in physical activity but also in social interactions and the mental health of children and young people (Wang et al., 2020). During the pandemic time, many video communication platforms were the main tool for teaching all types of classes (Vaimann et al., 2020). Therefore, children are using the Internet for school work and social interaction.

It should be emphasized that in our research, data were collected during a period (from November 2020 to March 2021) when the most restrictive policies were in place to prevent the spread of the virus, including the closure of primary and secondary schools, the cancellation of recreational team sports and activity classes for students, and the closure of public parks and playgrounds. Besides, the study was conducted after almost a year of online schooling, adjusting to new forms of learning, online classes, and studying alone at home without face-to-face interaction with teachers and classmates; this may have contributed to a lower perception of inclusion. The obtained results on the perception of students' inclusion in school education can be considered relatively high in comparison with the findings reported by Zurbriggen et al. (2017). As expected, in our research, it also turned out that the gender of the students did not differentiate their perception of inclusion in school education. We also found that respondents participating in remote learning caused by the coronavirus obtained statistically significantly lower results in the case of “emotional wellbeing in school” and a higher mean for the variable of “social relationships with other students” than the control group. These results can be considered very positive. It seems that the students managed to recognize the effectiveness of distant learning. Besides, the results for “academic competencies” may suggest that actions taken by teachers helped ensure that school learning was largely undisrupted. Tang et al. (2020) revealed that students were generally satisfied with life and 21.4% became more satisfied with life during school closures; the authors showed that children and adolescents perceived home quarantine as more positive than negative, and this yielded less psychological distress and more life satisfaction. The obtained results should be considered positive, taking into account the publications by other authors. It should also be emphasized that the self-perception of social inclusion is a stable phenomenon (Schwab et al., 2018). Besides, it was observed that social inclusion in the classroom was statistically significant enough to promote resilience (Ganotz et al., 2021), and students who feel personally accepted and integrated report higher satisfaction in life (Cruz-Ortiz et al., 2016; Moffa et al., 2016).

At the same time, it should be emphasized that the student's perception of inclusion in school education is influenced by many factors that are closely related to each other. Among them, special attention should be paid to participation in school physical activity. It seems reasonable to ask whether there are any correlations between the respondents' perception of inclusion in school education and their level of daily physical activity. Moreover, considerations regarding students' physical activity are also very important for other reasons. In recent years, many authors have pointed to the benefits of regular participation in physical activity, especially with regard to children and young people. For example, it is indicated that physical activity can help individuals achieve a positive self-concept and self-esteem and promote wellbeing in adolescents through the improvement of physical perceptions and body image (Rangul et al., 2012; Telles et al., 2013; Joseph et al., 2014; Goldfield et al., 2015; Das et al., 2016; Maugeri et al., 2020). In this context, it is easier for students to integrate with their peers and they can feel more involved in the education process (Fernández-Bustos et al., 2019; Shahidi et al., 2020). Physical activity has also positive effects in preventing and alleviating depressive symptoms (Schuch et al., 2016) and anxiety (Stubbs et al., 2017).

There is no doubt that all the points mentioned earlier are of particular importance in the setting of research results during the pandemic. The prevalence of depressive symptoms was 22.6% in primary school students (Xie et al., 2020) and 26.3% in secondary school students (Tang and Ying, 2020). The prevalence of anxiety symptoms in China equaled 18.9, 22.0, and 29.8% in primary school students from the Hubei Province (Xie et al., 2020), in primary and secondary school students from the Shaanxi Province (Li et al., 2020), and in secondary school students from the Sichuan Province (Tang and Ying, 2020), respectively. In turn, in previous pandemics, such as that of SARS, parents and children who were in quarantine presented with 4 times higher stress scores than the ones who were not in quarantine (Sprang and Silman, 2013). Therefore, it seemed reasonable and important to investigate Polish students.

Our research showed that only the level of walk MET (*p* = 0.75) differentiated girls and boys. A higher mean was observed in the group of female students. These results do not coincide with the findings by Bergier B. et al. (2012) and Bergier et al. (2012) or Bergier and Ignatjeva (2017). However, they are partly in line with studies by Groffik et al. (2019) and Mata et al. (2021). In addition, our results corroborate the observation that low-intensity activities (walking) are preferred among girls, and moderate- and high-intensity efforts among boys (Armstrong and Welsman, 2006). Besides, Dunton et al. (2020) indicated children's increased usage of local streets and sidewalks for physical activity during the COVID-19 pandemic, which was confirmed by parents who reported that their children spent more time going for a walk.

What draws attention is the level of total physical activity, which was higher in our analyses than in studies conducted by other authors (Pearson et al., 2009; Li et al., 2010; Bergier B. et al., 2012; Bergier et al., 2012; Van Stralen et al., 2014; Bergier and Ignatjeva, 2017).

In addition, our research found that the recommendations for vigorous physical activity were achieved by 37.78% of boys and 34.85% of girls. In turn, 69.70% of female students and 77.78% of male respondents met the recommendations for medium physical activity. It was also noted that 87.88% of girls and 86.67% of boys participating in the research fulfilled the recommendations for total physical activity. Therefore, considering the findings of other authors (Groffik et al., 2019), our results can be considered quite optimistic. Besides, Mavilidi et al. (2020) indicated that according to global estimates, fewer than 19% of young people met the recommended guidelines of 60 min per day of moderate-to-vigorous physical activity. Research among Australian biomedical students revealed physical activity levels reduced by 30% compared with the pre-pandemic years (Gallo et al., 2020). Besides, Castañeda-Babarro et al. (2020) observed that young participants (aged 18–24 years) had the highest decrease in moderate activity and walking time. In addition, disturbing results are available that revealed an increase in the childhood obesity rate by 2.4%, with a modestly higher impact in specific populations (An, 2020; Ricci et al., 2020; Hammami et al., 2022). Maugeri et al. (2020) also showed that total physical activity significantly decreased during quarantine in young people aged <21 years. In turn, Dunton et al. (2020) indicated that early in the pandemic period, children spent their free time on sedentary rather than physical activities. The authors also revealed a longer time devoted to sedentary behaviors among girls and older children than in boys and younger children. According to Shahidi et al. (2020), the activities available to children during the pandemic, especially in the context of quarantine and social distancing measures, are restricted. However, even with the restrictions of limited space or lack of special equipment, reaching the WHO recommendations is still achievable, even at home during self-isolation (Mata et al., 2021). Besides, taking into account that consuming unhealthy food, eating out of control, the number of snacks between meals, late-night snacking, and the numbers of main meals were significantly higher during the home confinement (Ammar et al., 2020, 2021), reaching the higher end of the recommendations is very valuable. It is also suitable to refer to the studies which showed that the most common physical activities during the early COVID-19 period were free to play and unstructured activity, e.g., running around (90% of children) and going for a walk (55% of children) (Dunton et al., 2020). Dunton et al. (2020) reported that children engaged in about 90 min of school-related sitting and over 8 h of leisure-related sitting a day, but parents of older children (aged 9–13 years) vs. younger children (aged 5–8 years) perceived greater decreases in physical activity and greater increases in sedentary behaviors between the pre- and early COVID-19 periods. Children were more likely to perform physical activity at home or on neighborhood streets during the early vs. the pre-COVID-19 periods (Dunton et al., 2020). Our results, as well as the results obtained by the authors mentioned earlier, are partly in line with another research conducted which showed that the PA levels and total weekly energy expenditure decreased during COVID-19 restrictions (Giustino et al., 2020; Gjaka et al., 2021). It was found that higher decreases in MET min/week during isolation were observed among males, adolescents, young adults, respondents with the overweight, and participants living in the city (Gjaka et al., 2021). Giustino et al. (2020) observed that the male group decreased the PA level more than the female one. In addition, the authors mentioned earlier found that respondents with the overweight showed the lowest level of PA during quarantine.

Our analyses revealed only negligible and low positive correlations in the whole sample, as well as in the female and male groups. In relation to the whole sample, we observed a low positive correlation between BMI and “academic competencies” and a negligible relationship not only between “vigorous MET” and “academic competencies” but also between ‘vigorous MET' and “sum PIQ score.” Among girls, only one low positive correlation was reported between BMI and “academic competencies.” In turn, in the group of boys, it was noticed that “housework MET” exhibited a low positive relationship with “social relationships with other students” and “sum PIQ score.” In addition, “medium MET” presented a low positive correlation with “academic competencies” and “sum PIQ score.”

It is worth mentioning that Ahmadi and Ahmadi (2020) proved that parental involvement in children's schooling was directly associated with the students' life satisfaction. Moreover, the sense of fairness and parental involvement was indirectly related to life satisfaction through belonging to the school, and teacher–student relationship exerted a significant indirect impact on life satisfaction through the sense of fairness and belonging to the school. It is also worth referring to research which indicates that increased levels of physical activity and reduced sedentary behavior might help satisfy basic psychological needs (e.g., social connectedness, self-acceptance, and perception of purpose in life) and, consequently, improve overall mental health in young people (Lubans et al., 2016; Rodriguez-Ayllon et al., 2019).

## Conclusion

This paper presents the first study concerning the effects of social isolation and learning online caused by the COVID-19 pandemic on the perception of being included in school education. The key strengths of the research are the typology of the study we appropriately selected during this period and its ease of access for the participants. In fact, due to the quarantine, it seems that the online survey is an ideal research instrument.

Our research observes that the students' gender did not differentiate their perception of inclusion in school education. The respondents participating in research during the COVID-19 pandemic obtained statistically significantly lower results in the case of “emotional wellbeing in school” and higher results in relation to “social relationships with other students” than the control group. Besides, it turned out that the COVID-19 pandemic was not a factor differentiating students' results in “academic competencies.” In addition, only the level of walk MET differentiated girls and boys: higher results were reported among female students. Besides, the recommendations for vigorous physical activity were achieved by 37.78% of boys and 34.85% of girls. In turn, 69.70% of female students and 77.78% of male students met the recommendations for medium physical activity, whereas 87.88% of girls and 86.67% of boys achieved the recommendations for total physical activity.

It is surprising that in the case of the whole sample, the analyses revealed a low positive correlation between BMI and “academic competencies” and a negligible relationship between “vigorous MET” and “academic competencies,” as well as between “vigorous MET” and “sum PIQ score.” It also turned out that girls presented only one low positive correlation between BMI and “academic competencies'. In turn, in the male group, “housework MET” exhibited a low positive relationship with “social relationships with other students” and “sum PIQ score.” It was also observed that “medium MET” had a low positive correlation not only with “academic competencies” but also with “sum PIQ score.”

## Limitations

There is no doubt that some limitations of the study and the potential areas for expanding the research should be identified. First of all, it is advisable to involve many more respondents in further studies. However, despite this limitation, the study recruited a quite large sample of adolescents. Besides, the students with special needs also should participate in the analyses. One should emphasize that the study employed self-report measures; these, although appropriate for evaluating subjective factors, could be supplemented with other tools in future investigations. It seems that assessing physical activity by using, for example, pedometers would be a good solution. Furthermore, the results of studies carried out during the pandemic were compared with the data collected in the years 2018–2019 among different respondents. In addition, it would be advisable to perform parallel analyses after the schools reopen. In this respect, one cannot estimate the perception of inclusion in school education or physical activity. A crucial point is that the psychological impact of quarantine can be observed even several months or years later on (Jeong et al., 2016; Liu et al., 2020). A further limitation is inherent to the design, as the study variables were only assessed by the students. Thus, only one dimension of the perception of inclusion was explored. Consequently, if the instrument is used by multiple informants, students, parents, and teachers could discuss the outcomes and investigate why a student feels unhappy at school and what each of the informants can do to improve the situation. This study was intended to show that the problem should be considered and thus provide an important indication for the inclusive debate. Among the possible areas of expanding this research project, performing longitudinal studies with the application of a cross-sectional and sequential analysis design seems justified. The aim would be to determine changes in the students' perception of inclusion in the school education process and physical activity over a given period; e.g., after 2–3 years. It seems that further longitudinal research should be performed to observe students' development in social contexts (behavior, competencies, and participation) and to develop beneficial inclusive education programs. Good inclusive education should certainly involve numerous social learning opportunities and interactions concentrated on all students' participation. Taking into account the above issues, a longitudinal study with follow-up surveys is needed to clarify the discussed relationships. Besides, the quantified influence of COVID-19 on physical activity will depend on the duration of the lockdown and the period of re-introducing the “normal life” settings in each country. It is, therefore, impossible to compare the obtained results with similar studies carried out in other countries. Bearing this in mind, further investigations on the subject and the obtained conclusions will turn out wide-ranging and of considerable value.

## Data Availability Statement

The raw data supporting the conclusions of this article will be made available by the authors, without undue reservation.

## Ethics Statement

The studies involving human participants were reviewed and approved by the Bioethics Committee of the Academy of Physical Education in Katowice. Written informed consent to participate in this study was provided by the participants' legal guardian/next of kin.

## Author Contributions

KK: conceptualization, data curation, formal analysis, methodology, resources, and writing—original draft. AZ: methodology, resources, and review and editing supervision. MZ: methodology and writing—review and editing. All authors contributed to the article and approved the submitted version.

## Funding

This research was funded by The Jerzy Kukuczka Academy of Physical Education in Katowice, grant number AWF/INS/ZB2/2022.

## Conflict of Interest

The authors declare that the research was conducted in the absence of any commercial or financial relationships that could be construed as a potential conflict of interest.

## Publisher's Note

All claims expressed in this article are solely those of the authors and do not necessarily represent those of their affiliated organizations, or those of the publisher, the editors and the reviewers. Any product that may be evaluated in this article, or claim that may be made by its manufacturer, is not guaranteed or endorsed by the publisher.

## Footnotes

^1^http://piqinfo.ch/wp-content/uploads/2021/01/PIQ-Polski_2021.pdf (elaborated by Dr Edyta Tominska Conte and Anna Zwierzchowska).

^2^ https://sites.google.com/site/theipaq/questionnaire_links/IPAQ_English_self-admin_short.pdf?attredirects=0&d=1

^3^ https://docs.google.com/viewer?a=v&pid=sites&srcid=ZGVmYXVsdGRvbWFpbnx0aGVpcGFxfGd4OjE0NDgxMDk3NDU1YWRlZTM

^4^ http://www.indares.com/public/Default.aspx
